# Center-surround interactions in a network model of layer 4Cα of primary visual cortex

**DOI:** 10.1186/1471-2202-14-S1-P435

**Published:** 2013-07-08

**Authors:** Christoph Metzner, Achim Schweikard, Bartosz Zurowski

**Affiliations:** 1 Institute for Robotics and Cognitive Systems, University of Luebeck, 23538 Luebeck, Germany; 2 Graduate School for Computing in Medicine and Life Sciences, University of Luebeck, 23538 Luebeck, Germany; 3 Department of Psychiatry, University Clinics Schleswig-Holstein, 23538 Luebeck, Germany

## 

Context integration is an ubiquitious principle in cortical processing underlying many perceptual and cognitive functions. Several neuropsychiatric disorders have been associated with an impairment of integration of context information, particularly schizophrenia [5]. One way to investigate the mechanisms underlying context processing and its impairments, is to look at context integration in the well-understood visual system. Center-surround interactions (CSI), i.e. the mutual influencing of stimuli presented in the center and in the surround of the visual or receptive field, respectively, are well established, both in animal neurophysiology and human psychophysical and neuroimaging studies.

We investigated orientation-selective center-surround interactions in a network model of the cortical layer 4Cα, the input layer of V1 and the first cortical stage where CSI effects occur. The model is based on a previous one [6] and consists of 1024 excitatory and 256 inhibitory multi-compartment cells coupled via AMPA, NMDA and GABA synapses with a virtual 44 × 44 layer of lateral geniculate nucleus (LGN) processing units. First, we used moving sinusoidal gratings of varying orientation (0°-330°, step size: 30°) to determine orientation selectivity of the neurons [4]. Second, we measured non-specific center-surround suppression by increasing the size of the grating at the preferred orientation and calculating the suppression index (SI) according to [1]. Finally, we measured orientation-specific surround suppression by introducing a second grating surrounding the optimally sized center grating. We varied the orientations of the surround grating and measured the SI for each orientation.

We found orientation and direction selectivity comparable to findings from neurophysiological studies[2,4] and those from another modeling study [4]. A high proportion (78%) of the model neurons show non-specific suppression with a mean SI of 47.21, which is comparable to animal studies in monkeys [1,3]. The orientation-selective suppression is strongest when the surround has the same orientation as the center grating and reduces with increasing angle deviation (mean SI parallel: 47.21; mean SI orthogonal: 36.72). This is in agreement with animal experiments [1] but also with human psychophysical studies [5,7].

We have presented a neuronal network model that showed non-specific and orientation-specific center-surround suppression in accordance with experimental and psychophysical data. Our model allows for investigating in detail the mechanisms supporting center-surround suppression and context-integration. This comprises changes of the perceptual input, characteristics of neurons and synapses as well as modulatory influences of GABA, for instance. Finally, our model is suited to systematically model the neural subtsrates of context integration impairments in neuropsychiatric disorders.
